# Ochratoxin A in Beers Marketed in Portugal: Occurrence and Human Risk Assessment

**DOI:** 10.3390/toxins12040249

**Published:** 2020-04-12

**Authors:** Liliana J. G. Silva, Ana C. Teixeira, André M. P. T. Pereira, Angelina Pena, Celeste M. Lino

**Affiliations:** LAQV, REQUIMTE, Laboratory of Bromatology and Pharmacognosy, Faculty of Pharmacy, University of Coimbra, Polo III, Azinhaga de Stª Comba, 3000-548 Coimbra, Portugal; catarina_tex@hotmail.com (A.C.T.); andrepereira@ff.uc.pt (A.M.P.T.P.); apena@ci.uc.pt (A.P.); cmlino@ci.uc.pt (C.M.L.)

**Keywords:** ochratoxin A, beer, immunoaffinity clean-up, LC-FD, human risk assessment

## Abstract

Ochratoxin A (OTA) is produced by fungi present in several agricultural products with much relevance to food safety. Since this mycotoxin is widely found in cereals, beer has a potential contamination risk. Therefore, it was deemed essential to quantify, for the first time, the levels of OTA in beer, a cereal-based product that is marketed in Portugal, as well as to calculate the human estimated weekly intake (EWI) and risk assessment. A total of 85 samples were analyzed through immunoaffinity clean-up, followed by liquid chromatography-fluorescence detection (LC-FD). This analytical methodology allowed a limit of quantification (LOQ) of 0.43 µg/L. The results showed that 10.6% were contaminated at levels ranging between <LOQ and 11.25 µg/L, with an average of 3.14 ± 4.09 µg/L. Samples of industrial production presented lower incidence and contamination levels than homemade and craft beers. On what concerns human risk, the calculated EWI was significantly lower than the tolerable weekly intake (TWI). However, in the worst case scenario, based on a high concentration, the rate EWI/TWI was 138.01%.

## 1. Introduction

Mycotoxins, secondary metabolites produced by fungi, affect 25% of crops worldwide [1]. Mycotoxin contamination usually takes place during crop growth due to adverse environmental conditions, inappropriate harvesting, storage, or processing procedures [2].

Ochratoxin A (OTA), the major mycotoxin of the ochratoxins’ group and the one presenting great toxicological concern, is produced by *Penicillium verrucosum*, *Aspergillus ochraceus*, and rarely by some strains of *Aspergillus niger* [3]. OTA has been reported as nephrotoxic, carcinogenic, teratogenic, genotoxic, and immunotoxic. This mycotoxin also disturbs blood coagulation, hinders protein synthesis, endorses cell membrane peroxidation, abolishes calcium homeostasis, and constrains mitochondrial respiration [4]. In addition, it is epidemiologically associated to the human Balkan endemic nephropathy (BEN) disease and to urinary tract tumors [5,6]. Moreover, it is described as a cumulative mycotoxin since it is easily assimilated by the digestive tract and is gradually excreted by the urinary system [4]. Since 1993, OTA has been described as a possible carcinogen to humans, group 2B, by the International Agency for Research on Cancer (IARC) [7].

OTA generally occurs in numerous food products. This includes cereals, oleaginous seeds, green coffee, wine, meat, cocoa, spices, and fruit berries, which are contaminated at levels that vary according to environmental and processing conditions [8,9]. Moreover, some studies report that the prevalence and levels of OTA in organic cereals is higher when compared to non-organic cereals [10].

Due to the fact that OTA widely occurs in cereals, beer, which is a cereal product, has a potential contamination risk [11]. OTA was first reported in beer in 1983 [12]. Since then, several analytical methodologies were developed to study the natural incidence of OTA in this beverage. Most studies employed solid phase extraction through immunoaffinity columns (IAC) [12,13,14,15,16,17]. For detection and quantification, most authors employed liquid chromatography with fluorescence detection (LC-FD) [8,13,14,18,19], with limits of detection (LODs) varying between 0.002 µg/L and 1 µg/L. Liquid chromatography with tandem mass detection (LC-MS-MS) or ultra-pressure liquid chromatography with mass detection (UPLC-MS) was also applied, with LODs varying between 0.75 and 0.0003 µg/L, respectively [12,20].

Worldwide, several studies have investigated the presence of OTA in beer. Its occurrence was reported in Brazil [14], South Africa [18], Iran [21,22], Turkey [23], China [24], Japan [25], and Europe [13,20]. Namely, European studies reported it in Germany [26], Belgium [17], Spain [2], Italy [21,27], and the Czech Republic [19,28].

According to the scientific literature, the occurrence of OTA in beer samples is usually at low levels. In these studies, the minimum levels found ranged between 0.0009 and 2.7 μg/L, in Iran and Europe, respectively [20,22]. Some studies reported higher concentrations, up to 18 μg/L in Brazil [14]. However, one of the studies showed significantly higher values, up to 2340 μg/L [18].

The incidence of OTA in beer depends on the contamination of brewing materials, such as barley and barley malt, with ochratoxigenic fungi species [12]. During the production of beer, considerable OTA losses (40–89%) have been perceived in the grist during mashing, most possibly owing to proteolytic degradation [11]. Another 16% may be eliminated with spent grains [11]. With the fermentation process, OTA decreases in the range of 2% to 69% [11]. The remaining OTA passes on to the final beer product [12].

This contaminant may eventually reach consumers, and a frequent consumption of contaminated products could suppose a risk for human health [20]. There is no maximum allowable limit established by the European Commission (EC) for OTA content in beer [12]. Although there is no defined limit for beer, a maximum of 3 μg/kg for malt has been established by the European Union [29]. However, there are guideline levels established by the Netherlands (0.5 μg/L), Finland (0.3 μg/L), and Italy (0.2 μg/L) [8]. A tolerable weekly intake (TWI) of 120 ng/kg body weight (b.w.) was set for OTA in 2006 by the European Food Safety Authority (EFSA) [30].

Our main goal was to verify, for the first time, the OTA contamination of the beer marketed and consumed in Portugal, as well as to calculate the human estimated daily intake and risk assessment.

## 2. Results and Discussion

### 2.1. Validation and Quality Control

Validation and quality control fulfilled the European guidelines [31] and the results are presented in Table 1.

Linearity results, both on standard and matrix-matched assays, were suitable, with correlation coefficients (*r*^2^) of 0.998 and 0.999, respectively. The obtained limit of detection (LOD) and limit of quantification (LOQ) were 0.14 µg/L and 0.43 µg/L, respectively. The value of matrix effect (ME) obtained was considered negligible, 109%.

Accuracy and precision were adequate. Recoveries varied from 81.0% to 86.0%. Intra-day repeatability ranged from 1.72% to 2.43% and inter-day repeatability ranged from 2.74% to 6.25%. Both accuracy and precision results were adequate according to the requirements established by the EC 401/2006 directive [31].

### 2.2. Frequency and Occurrence of OTA in Beer Samples

In the present study, from the 85 analyzed beers, nine samples (10.6%) were contaminated at concentrations ranging from < LOQ to 11.25 μg/L, with an average level of 3.14 ± 4.09 µg/L (median of 1.74%). One of the contaminated samples presented a concentration between the LOD and the LOQ. Therefore, for results’ analysis, half of the LOQ value was considered (Table 2).

Among the contaminated samples, three contained maize, two contained wheat, and the other four indicated only the presence of barley and/or barley malt. The concentrations found in samples containing maize were 1.22, and 0.54, μg/L. In samples containing wheat, the contamination values were similar, 1.81 μg/L and 0.50 μg/L. In samples that indicated the presence of barley alone, the values were higher, at 1.81, 1.74, 9.21, and 11.25 μg/L, with the latter two being homemade beer samples. Samples with just barley in their composition represented 44.4% of the contaminated samples (Table 3). A similar higher percentage was found in samples with mixed cereals (55.5%). The difference in the OTA levels was 6.00 μg/L (median 5.51 μg/L) versus 0.86 μg/L (median 0.54 μg/L), which was due to the high levels found in the two homemade samples that were analyzed. Regarding these homemade samples, a limited number was possible to achieve. Although these are preliminary results, we thought it was interesting to include them in the study.

Although there is evidence that organic foods often contain high concentrations of natural toxins produced by fungi [10], whereas conventional foods tend to contain more synthetic compounds such as pesticide residues, none of the contaminated samples were of organic origin. In addition, the use of fungicides or preservatives in insufficient amounts can lead to a more serious situation due to stress caused in fungi, which leads to a stimulation of mycotoxin production [32].

Regarding the type of production, homemade beers presented the highest contamination levels at 10.23 ± 1.44 μg/L. Industrial and craft beers showed higher frequencies at 44.4% and 33.3%, but lower mean levels which were measured at 1.25 ± 0.74 μg/L (median 1.48 μg/L) and 0.95 ± 0.66 μg/L (median 0.54 μg/L), respectively.

Two homemade samples showed really high levels, with 9.21 μg/L and 11.25 μg/L. Given that the maximum level of OTA is not currently established for beer but taking into account the limit established for wine (2 μg/L) (EC No. 1881/2006), these two samples exceeded at least 4.5 times and 5.5 times, respectively, this limit. Cereals used in homemade brewing are sold in plastic packaging presenting a greater risk of inadequate storage due to the moisture content, which favors their contamination. Another reason is that these types of beers are not subject to rigorous quality control and the cereals used are not as strictly controlled as those used in craft/industrial brewed beers.

Mycotoxins are highly stable and able to resist high temperatures and pH levels. The procedures involved in beer production use maximum operation temperatures lower than those able to destroy mycotoxins. However, they may impact mycotoxin levels given the physical, chemical, and biochemical alterations that take place [33]. The presence of OTA has been associated with barley malt contamination with ochratoxigenic species, namely *Penicillium verrucosum*. The OTA produced in the grains passes on to the wort and, although fermentation decreases its levels, the toxin is not completely removed [34]. Steeping, kilning, mashing, fermentation, and clarification are the most important steps of beer production presenting a negative impact on mycotoxins’ levels. During these phases, mycotoxins may be removed through the drainage water, with the spent grains or with the fermentation residue. Moreover, they can also be diluted or destroyed as a result of the thermic treatment [33].

In the present study, every contaminated sample was pale beer. Drying (kilning) conditions are unfavorable for fungi (especially the first phase with a temperature of 50 °C and grain moisture of 45%). Knowing that the intensity of kilning and roasting (if applied) is crucial in malt flavor and color formation [33], fungi are removed during this process but thermostable mycotoxins produced before kilning persist [35]. Nowadays, the largest percentage of malt in most beers is pale malt which is only mildly dried at moderate temperature and might explain our results. Lager beers presented a higher contamination frequency (55.5%) but lower mean levels 0.86 ± 0.65 µg/L (median 0.54 μg/L) when compared to ale beers, the latter of which presented a frequency of 44.4% and 6.00 ± 4.95 µg/L (median 3 μg/L) mean contamination levels. On the contrary to Mateo et al. [34], a longer fermentation process and, consequently, a higher alcohol content, did not reduced the OTA level.

The studied samples were purchased from different retail outlets located in Coimbra (Portugal) but originated from 10 different countries. It was observed that from the nine contaminated samples, six (66.7%) were produced in Portugal, which provides evidence for the necessity of a greater control. Nonetheless, the mean levels found in the Portuguese samples which were 1.02 ± 0.70 µg/L (median 0.88 μg/L), were lower than in those from abroad.

The levels found in the present study are similar to those of other studies carried out in different countries. According to the scientific reported literature, the mean OTA content varies from 0.02 µg/L to 1.47 µg/L, for the samples analyzed in Turkey [23] and Germany [26], respectively. The minimum and maximum values vary between 0.012–0.045 µg/L and 1.5–2,34 µg/L in samples from Turkey [23] and South Africa [18], respectively. More recently, in 2017 Peters et al. reported the presence of mycotoxins, including OTA, in more than 1000 beers collected from 47 countries. OTA was found in five samples from Norway and England, ranging from 0.3 to 0.6 μg/L [36]. Bertuzzi et al. found OTA in the most sold beers in Italy with a mean concentration of 0.007 μg/L, with a maximum value of 0.07 μg/L [37].

With regard to the incidence of contamination, a frequency of 100% was found in samples collected in countries such as Spain [15], Hungary [2], Italy [27], Iran [22], Germany [26], and the Czech Republic [19]. The lowest OTA frequency was found in countries such as Brazil (0–5.3%) [14], China (0%) [24], Korea [16] and Turkey (14%) [23]. Recently, OTA was found in 45.8% of the most sold beers in Italy [37]. 

### 2.3. Human Estimated Daily Intake and Risk Assessment

The consumption of wine and beer make up part of the European culture. While Southern Europe is usually associated with wine consumption, Northern Europe is associated with beer. Nonetheless, since the 1960s wine consumption in Portugal has declined and beer is now the most consumed alcoholic beverage [38]. The number of brewing companies and the number of microbreweries increased, reflecting growth in the craft beer and specialty beer segment [39].

Three different scenarios were used to perform three EDI evaluations: the OTA contamination levels of the total analyzed samples (I); the mean OTA content regarding the nine positive samples; and the worst case scenario using the highest OTA level. In the first evaluation the EDI was 0.67 ng/kg b.w./day, in the second a value of 6.35 ng/kg b.w./day was obtained, and for the worst case scenario the value was 22.74 ng/kg b.w./day. When considering the estimated weekly intake (EWI) the obtained results for the three different scenarios were 4.71, 44.48, and 159.16 ng/kg b.w./week, respectively (Table 4).

For risk assessment, the most recent tolerable weekly intake (TWI) value established by EFSA [30] was used (120 ng/kg b.w./week). In the first evaluation scenario, the percentage of estimated weekly intake (EWI) versus TWI was 3.92%. In the second, it was 37.06%, and in the third scenario it was 132.63%. In the first two situations, the ingestion of OTA through the consumption of beer presents no risk to the respective consumers. The inverse situation was observed for the worst case scenario.

This risk evaluation has its limitations since it is based on consumption and occurrence data which contained a high number of negative samples in this study. Nonetheless, it is a contribution to assess the human risk posed by the consumption of beer.

In food monitoring studies, the driving force is often enforcement of legal limits [40]. Current legislation does not include limits for the occurrence of OTA in beer, but the identified concentrations, especially in homemade beers, should be considered.

## 3. Conclusions

The proposed analytical methodology, sample pre-treatment with sodium bicarbonate and PBS followed by IAC clean-up and LC-FD, enabled low detection and quantification levels and good results regarding accuracy and precision.

The application of this method to 85 samples showed that 10.59% were contaminated, two of which were homemade and presented considerable concentrations (9.21 and 11.25 μg/L). The discrepancy found can be explained by the storage of cereals already prepared for homemade brewing that may not be as strictly controlled as the others.

Three risk assessments were carried out based on three different scenarios. In the first two, the ingestion of OTA through the consumption of beer presents no risk to the respective consumers. The inverse situation was observed for the worst case scenario, where the most contaminated sample was considered.

The EU established maximum limits for OTA in some products. Current legislation does not include limits for the occurrence of OTA in beer, but the identified concentrations, especially in homemade beers, should be considered. For this reason, it is important to adopt preventive measures and develop control programs, reviewing the critical points where OTA production can occur in order to minimize human exposure to OTA.

## 4. Materials and Methods

### 4.1. Chemicals and Materials

HPLC grade acetonitrile and methanol and PBS tablets were purchased from Sigma Chemicals Co. (St. Louis, MO, USA). Toluene was acquired from Carlo Erba (Milan, Italy). Acetic acid was obtained from Merck (Darmstadt, Germany) and 98% purity degree OTA was obtained from Sigma Chemicals Co. (St. Louis, MO, USA).

The OTA stock solution was prepared at 250 µg/mL in toluene-acetic acid (99:1) and stored at −20 °C. The intermediate solution was prepared at 10 µg/mL, in mobile phase, and diluted accordingly to obtain the external calibration solutions.

Bi-distilled water was obtained from a Milli-Q System (Millipore, Bedford, MA, USA). A mixture of acetonitrile–water–acetic acid (49.5:49.5:1 *v*/*v*/*v*) was used as mobile phase. All chromatographic solvents were filtered through a 0.20 µm membrane filter (Whatman GmbH, Dassel, Germany) and degassed.

Immunoaffinity columns (IACs) OchraTest^TM^ (Vicam/Waters, Milford, MA, USA) were used for clean-up. Micro-glass fiber paper (150 mm, Munktell & Filtrak GmbH, Bärenstein, Germany), cellulose nitrate (0.45 µm, Sartorius Stedim Biotech GmbH, Göttingen, Germany), and Durapore membrane filter (0.22 µm, GVPP, Millipore, Ireland) were also used.

### 4.2. Sampling and Sample Characterization

In 2018, 84 bottled commercial beer samples and one draft beer, representing 59 brands, were randomly acquired from different retail outlets and supermarkets located in Coimbra (Coimbra, Portugal). The samples were classified based on the type of production, type of beer, color, fermentation, alcohol content, and country of origin.

In total, 61 samples were industrial manufactured, 21 were craft beers, and 3 were homemade beers. Regarding the type of beer, 44 were ale with top-fermentation, and 41 were lager, two of which were fruit/vegetable beers with bottom-fermentation. Of all the samples, 59 were of pale color, 2 were pale-red, and 24 were dark beers. Of the 85 beer samples, 30 were strong beers, with an alcohol content ≥6% and 55 contained alcohol <6% (3 samples were non-alcoholic, with <1% alcohol).

The majority of the samples (79) were of European origin and six were from abroad. Of all the beers, 36 were produced in Portugal. The imported beers originated from Belgium (n = 15), the Czech Republic (n = 3), Germany (n = 11), Ireland (n = 1), Mexico (n = 1), the Netherlands (n = 5), New Zealand (n = 2), Poland (n = 1), Russia (n = 3), Scotland (n = 2), and Spain (n = 5).

Among these samples, seven were brewed with organically produced materials and were labelled as organic beers. None of the samples was analyzed beyond their expiration date. Until analysis, they were stored in the dark at 4 °C and all the information available on the labels was assembled.

### 4.3. Experimental Procedure

Based on a previously reported analytical methodology [41], degassed and consecutively filtered beer samples (10 mL) were added, of 4% sodium bicarbonate (1.25 mL) and 10 mL of PBS. After centrifugation, the extract was loaded into an IAC cartridge for clean-up. After a washing step with 5 mL of water, OTA was eluted with 4 mL of methanol. Afterwards, the solvent was evaporated at 40 °C under a gentle nitrogen stream, and the dried residue was stored at −20 °C until analysis. For liquid chromatography with fluorescence detection (LC-FD), redissolution was accomplished with 1 mL of mobile phase. Following filtration through a Durapore membrane filter, 20 µL were injected into the HPLC system that consisted of a 805 manometric module Gilson, and a fluorimetric detector from Jasco (Tokio, Japan) FP-2020 Plus. Excitation and emission wavelengths were 336 nm and 440 nm, respectively. A C18 Nucleosil 5 µm (4.6 × 250 mm i.d.) column (Hichrom, Leicestershire, UK) was used and the flow rate was set at 1 mL min ^−1^. The total run time was 15 min.

### 4.4. Validation and Quality Control Assays

Validation and quality control assays were performed as set by European guidelines [31]. Different parameters were evaluated, including linearity, limit of detection (LOD) and limit of quantification (LOQ), matrix effect (ME), accuracy and precision. Linearity was assessed using standards (2.5–25 µg/L), and matrix-matched solutions (0.25–2.5 µg/L). Sensitivity was evaluated through the matrix-matched calibration curve.

The LOD was set as |3.3Sy/x|/b and the LOQ as |10Sy/x|/b, respectively, knowing that b corresponds to the slope and Sy/x corresponds to the residual standard deviation of the linear function.

The ME (%) corresponds to the percentage of the ratio of matrix-matched calibration curve slope (B) and the slope of the standard calibration curve (A). The results were interpreted as follows: 100% signifies an absence of ME; a result higher than 100% corresponds to a signal enhancement; and a result lower than 100% corresponds to a signal suppression.

Accuracy and precision were evaluated using blanks and fortified samples at three levels (0.5, 1.0, and 2.0 µg/L). Three replicates were made (n = 3), in three different days for each fortification level. The relative standard deviation (RSD) of intra-day and inter-day repeatability were assessed to evaluate the precision of the analytical methodology.

### 4.5. Calculation of the Human Estimated Daily Intake and Risk Assessment

A deterministic method [42] was used to calculate the OTA estimated daily intake (EDI) through the consumption of beer through the following equation (*1*):

EDI = (∑c).(CN ^−1^ D ^−1^ K ^−1^), (1)

where ∑c is the OTA sum in the positive samples (µg/L), C corresponds to the mean annual beer consumption estimated per inhabitant, N is the samples’ number, D corresponds to the days of a year, and K is the body weight (kg). According to Statistics Portugal (INE) data, the beer consumption in 2016 was 50.9 L/inhabitant (C) [43]. The mean body weight considered for the Portuguese adult population was 69 kg (K) [44].

Three different scenarios were used to perform three EDI evaluations. In the first scenario, the OTA contamination levels were taken into consideration for the total of all analyzed samples. For the second scenario, the mean OTA content was considered. Finally, the worst case scenario was observed using the highest OTA level.

## Figures and Tables

**Table 1:** Analytical quality control data obtained for OTA in beer spiked samples.

Spiking Level (µg/L)	Recovery (%)	RSD within-day (%)	RSD between-day (%)
0.5	81.0	2.43	2.74
1	86.0	2.36	5.26
2	85.0	1.72	6.25

**Table 2:** OTA contamination levels (µg/L) found in the positive samples.

**Sample**	**Production**	**Type**	**Color**	**Type of Cereal**	**Alcohol Content (%)**	**Origin**	**Contamination (µg/L)**
17	Craft beer	Ale	Pale	Barley and wheat malt	4.7	Portugal	1.81
20	Craft beer	Lager	Pale	Barley and wheat malt	5.1	Portugal	0.5
22	Craft beer	Lager	Pale	Barley malt, maize	6	Portugal	0.54
31	Homemade	Ale	Pale	Barley malt	4.2	New Zealand	9.21
32	Homemade	Ale	Pale	Barley malt	5.6	New Zealand	11.25
6	Industrial	Lager	Pale	Barley malt, maize, barley	5	Portugal	<LOQ
18	Industrial	Lager	Pale	Barley malt	5	Portugal	1.81
19	Industrial	Ale	Pale	Barley malt	8.5	Belgium	1.74
21	Industrial	Lager	Pale	Barley malt, maize, barley	5.2	Portugal	1.22
**Total**							
Frequency (%)	‒	‒	‒	‒	‒	‒	10.6
Range (µg/L)	‒	‒	‒	‒	‒	‒	<LOQ‒11.25
Mean ± SD (µg/L)	‒	‒	‒	‒	‒	‒	3.14 ± 4.09
Median							1.74

**Table 3:** Frequency (%), OTA mean, and median contamination levels (µg/L) in different categories of contaminated beer.

Category		Frequency Positive (total)	Mean ± SD	Median
Production	Industrial	44.4 (4.7)	1.25 ± 0.74	1.48
	Craft	33.3 (3.5)	0.95 ± 0.66	0.54
	Homemade	22.2 (2.4)	10.23 ± 1.44	10.23
Type of beer	Lager	55.5 (5.9)	0.86 ± 0.65	0.54
	Ale	44.4 (4.7)	6.00 ± 4.95	3
Alcohol (%)	<6%	77.8 (8.2)	3.72 ± 4.53	1.81
	≥6%	22.2 (2.4)	1.14 ± 0.85	1.14
Type of cereal	Barley	44.4 (4.7)	6.00 ± 4.95	5.51
	Mixture	55.5 (5.9)	0.86 ± 0.65	0.54
Origin	Portugal	66.7 (7.1)	1.02 ± 0.70	0.88
	Abroad	33.3 (3.5)	7.40 ± 5.00	9.21

**Table 4:** Estimated daily intake and risk assessment.

Scenarios	EDI (ng/kg b.w./day)	EWI (ng/kg b.w./week)	EWI/TWI *^d^* (%)
I *^a^*	0.67	4.71	3.92
II *^b^*	6.35	44.48	37.06
III *^c^*	22.74	159.16	132.63

*^a^* n = 85 samples. *^b^* n = 9 samples. *^c^* The most contaminated sample was considered. *^d^* TWI of 120 ng/kg b.w./week was considered [30].

## References

[1] Eskola M., Kos G., Elliott C.T., Hajšlová J., Mayar S., Krska R. (2019). Worldwide contamination of food-crops with mycotoxins: Validity of the widely cited ‘FAO estimate’ of 25%. Crit. Rev. Food Sci. Nutr..

[2] Soto J.B., Fernández-Franzón M., Ruiz M.J., Juan-García A. (2014). Presence of ochratoxin a (OTA) mycotoxin in alcoholic drinks from southern european countries: Wine and beer. J. Agric. Food Chem..

[3] Malir F., Ostry V., Pfohl-Leszkowicz A., Malir J., Toman J. (2016). Ochratoxin A: 50 years of research. Toxins.

[4] Duarte S.C., Pena A., Lino C.M. (2011). Human ochratoxin A biomarkers-from exposure to effect. Crit. Rev. Toxicol..

[5] Gifford F.J., Gifford R.M., Eddleston M., Dhaun N. (2017). Endemic Nephropathy around the World. Kidney Int. Rep..

[6] Jadot I., Declèves A.E., Nortier J., Caron N. (2017). An integrated view of aristolochic acid nephropathy: Update of the literature. Int. J. Mol. Sci..

[7] Ostry V., Malir F., Toman J., Grosse Y. (2017). Mycotoxins as human carcinogens—The IARC Monographs classification. Mycotoxin Res..

[8] Aresta A., Palmisano F., Vatinno R., Zambonin C.G. (2006). Ochratoxin A determination in beer by solid-phase microextraction coupled to liquid chromatography with fluorescence detection: A fast and sensitive method for assessment of noncompliance to legal limits. J. Agric. Food Chem..

[9] Juan C., Mañes J., Font G., Juan-García A. (2017). Determination of mycotoxins in fruit berry by-products using QuEChERS extraction method. LWT Food Sci. Technol..

[10] Juan C., Moltó J.C., Lino C.M., Mañes J. (2008). Determination of ochratoxin A in organic and non-organic cereals and cereal products from Spain and Portugal. Food Chem..

[11] Anli E., Alkis İ.M. (2010). Ochratoxin A and Brewing Technology: A Review. J. Inst. Brew..

[12] Běláková S., Benešová K., Mikulíková R., Svoboda Z. (2011). Determination of ochratoxin A in brewing materials and beer by ultra performance liquid chromatography with fluorescence detection. Food Chem..

[13] Bertuzzi T., Rastelli S., Mulazzi A., Donadini G., Pietri A. (2011). Mycotoxin occurrence in beer produced in several European countries. Food Control.

[14] Kawashima L.M., Vieira A.P., Valente Soares L.M. (2007). Fumonisin B 1 and ochratoxin A in beers made in Brazil Fumonisina B 1 e ocratoxina A em cervejas fabricadas no Brasil. Ciência E Tecnol. Aliment..

[15] Medina Á., Jiménez M., Gimeno-Adelantado J.V., Valle-Algarra F.M., Mateo R. (2005). Determination of ochratoxin A in beer marketed in Spain by liquid chromatography with fluorescence detection using lead hydroxyacetate as a clean-up agent. J. Chromatogr. A.

[16] Park J.W., Chung S.H., Kim Y.B. (2005). Ochratoxin a in Korean food commodities: Occurrence and safety evaluation. J. Agric. Food Chem..

[17] Tangni E.K., Ponchaut S., Maudoux M., Rozenberg R., Larondelle Y. (2002). Ochratoxin A in domestic and imported beers in Belgium: Occurence and exposure assessment. Food Addit. Contam..

[18] Odhav B., Naicker V. (2002). Mycotoxins in South African traditionally brewed beers. Food Addit. Contam..

[19] Lhotská I., Šatínský D., Havlíková L., Solich P. (2016). A fully automated and fast method using direct sample injection combined with fused-core column on-line SPE-HPLC for determination of ochratoxin A and citrinin in lager beers. Anal. Bioanal. Chem..

[20] Rubert J., Soler C., Marín R., James K.J., Mañes J. (2013). Mass spectrometry strategies for mycotoxins analysis in European beers. Food Control.

[21] Prelle A., Spadaro D., Denca A., Garibaldi A., Gullino M.L. (2013). Comparison of clean-up methods for ochratoxin a on wine, beer, roasted coffee and chili commercialized in Italy. Toxins.

[22] Mahdavi R., Khorrami S.A.H., Jabbari V. (2007). Evaluation of ochratoxin A contamination in non alcoholic beers in Iran. Res. J. Biol. Sci..

[23] Kabak B. (2009). Ochratoxin A in cereal-derived products in Turkey: Occurrence and exposure assessment. Food Chem. Toxicol..

[24] Wu J., Tan Y., Wang Y., Xu R. (2011). Occurrence of ochratoxin a in wine and beer samples from China. Food Addit. Contam. B Surveill..

[25] Tamura M., Uyama A., Mochizuki N. (2011). Development of a Multi-mycotoxin Analysis in Beer-based Drinks by a Modified QuEChERS Method and Ultra-High-Performance Liquid Chromatography Coupled with Tandem Mass Spectrometry. Anal. Sci..

[26] Reinsch M., Töpfer A., Lehmann A., Nehls I., Panne U. (2007). Determination of ochratoxin A in beer by LC-MS/MS ion trap detection. Food Chem..

[27] Novo P., Moulas G., França Prazeres D.M., Chu V., Conde J.P. (2013). Detection of ochratoxin A in wine and beer by chemiluminescence-based ELISA in microfluidics with integrated photodiodes. Sens. Actuators B Chem..

[28] Ostry V., Malir F., Dofkova M., Skarkova J., Pfohl-Leszkowicz A., Ruprich J. (2015). Ochratoxin a dietary exposure of ten population groups in the czech republic: Comparison with data over the world. Toxins.

[29] European Commission (2006). Commission Regulation (EC) No 1881/2006. Off. J. Eur. Union.

[30] EFSA (2006). Opinion of the Scientific Panel on Contaminants in the food Chain on a Request from the Commission Related to Ochratoxin A in Food. Efsa J..

[31] European Commission (2006). Commission Regulation (EC) No 401/2006 of 23 February 2006 laying down the methods of sampling and analysis for the official control of the levels of mycotoxins in foodstuffs. Off. J. Eur. Union.

[32] Harcz P., Tangni E.K., Wilmart O., Moons E., Van Peteghem C., De Saeger S., Schneider Y.J., Larondelle Y., Pussemier L. (2007). Intake of ochratoxin A and deoxynivalenol through beer consumption in Belgium. Food Addit. Contam..

[33] Pascari X., Ramos A.J., Marín S., Sanchís V. (2018). Mycotoxins and beer. Impact of beer production process on mycotoxin contamination. A review. Food Res. Int..

[34] Mateo R., Medina Á., Mateo E.M., Mateo F., Jiménez M. (2007). An overview of ochratoxin A in beer and wine. Int. J. Food Microbiol..

[35] Mastanjević K., Šarkanj B., Krska R., Sulyok M., Warth B., Mastanjević K., Šantek B., Krstanović V. (2018). From malt to wheat beer: A comprehensive multi-toxin screening, transfer assessment and its influence on basic fermentation parameters. Food Chem..

[36] Peters J., Van Dam R., Van Doorn R., Katerere D., Berthiller F., Haasnoot W., Nielen M.W.F. (2017). Mycotoxin profiling of 1000 beer samples with a special focus on craft beer. PLoS ONE.

[37] Bertuzzi T., Rastelli S., Mulazzi A., Donadini G., Pietri A. (2018). Known and Emerging Mycotoxins in Small- and Large-Scale Brewed Beer. Beverages.

[38] Silva A.P., Jager G., Zyl H., Van Voss H., Hogg T., Graaf C., De Patricia A., Jager G., Zyl H., Van Voss H. (2017). Cheers, proost, saúde: Cultural, contextual and psychological factors of wine and beer consumption in Portugal and in the Netherlands. Crit. Rev. Food Sci. Nutr..

[39] The Brewers of Europe (2016). The Contribution Made by Beer to the European Economy.

[40] De Nijs M., Mengelers M.J.B., Boon P.E., Heyndrickx E., Hoogenboom L.A.P., Lopez P., Mol H.G.J. (2016). Strategies for estimating human exposure to mycotoxins via food. World Mycotoxin J..

[41] Anselme M., Tangni E.K., Pussemier L., Motte J.C., Van Hove F., Schneider Y.J., Van Peteghem C., Larondelle Y. (2006). Comparison of ochratoxin A and deoxynivalenol in organically and conventionally produced beers sold on the Belgian market. Food Addit. Contam..

[42] IPCS (2009). Dietary exposure assessment of chemicals in food. Principles and Methods for the Risk Assessment of Chemicals in Food.

[43] INE Statistics Portugal (2017). Balança Alimentar Portuguesa 2012–2016.

[44] Arezes P.M., Barroso M.P., Cordeiro P., Costa L.G., Miguel A.S. (2006). Estudo Antropométrico da População Portuguesa.

